# Gut microbiota in anemia: mechanistic insights into iron metabolism, vitamin synthesis, and immune regulation

**DOI:** 10.3389/fimmu.2026.1900845

**Published:** 2026-07-23

**Authors:** Lanxin Xu, Ying Gao, Yang Li, Zunsong Wang

**Affiliations:** 1 Department of Nephrology, The First Affiliated Hospital of Shandong First Medical University & Shandong Provincial Qianfoshan Hospital, Shandong Institute of Nephrology, Jinan, Shandong, China; 2 Department of Geriatric Endocrinology, The Fifth People’s Hospital of Jinan, Jinan, Shandong, China

**Keywords:** anemia, gut microbiota, immune modulation, iron metabolism, vitamin B12

## Abstract

The gut microbiota, as a vital micro-ecological system within the human body, plays a crucial role in regulating diverse physiological functions. Recent research has increasingly demonstrated its close association with the occurrence and progression of anemia. This review summarizes current understanding of how the gut microbiota influences iron metabolism, vitamin synthesis—particularly vitamin B12—and immune modulation, all of which are key factors in the pathogenesis of anemia. We explore the mechanisms by which dysbiosis of the gut microbiota contributes to anemia development, including disruptions in nutrient absorption and inflammatory responses. Furthermore, we analyze recent clinical studies that investigate the relationship between gut microbiota alterations and different anemia subtypes. By integrating the latest basic and clinical research findings, this review aims to provide a comprehensive overview of the gut microbiota’s role in anemia and to highlight its potential as a novel therapeutic target. The insights offered here may guide future research and clinical interventions focused on microbiota modulation as an innovative strategy for anemia management.

## 1 Introduction

The human gut harbors a vast and diverse microbial community, collectively termed the gut microbiota, which plays an indispensable role in host physiology. This complex ecosystem participates actively in nutrient absorption, metabolic regulation, and immune homeostasis, thereby influencing systemic health (1–4). The gut microbiota’s capacity to modulate host iron metabolism has garnered increasing attention, particularly given iron’s critical role in erythropoiesis and oxygen transport via hemoglobin. Disruptions in this finely balanced host-microbiota interplay can contribute to hematological disorders such as anemia, characterized by decreased hemoglobin concentration and impaired oxygen delivery to tissues (5, 6). In this review, “gut microbiota dysbiosis” refers to a state of imbalance in microbial community structure and function, typically characterized by reduced microbial diversity, depletion of beneficial commensal bacteria (e.g., Bifidobacterium and Faecalibacterium), and enrichment of potentially pathogenic or pro-inflammatory taxa (e.g., Enterobacteriaceae). In addition to compositional changes, dysbiosis also encompasses functional alterations, including disrupted short-chain fatty acid production, impaired vitamin biosynthesis, and increased endotoxin production. Anemia remains a prevalent clinical condition worldwide, manifesting as a common symptom across diverse patient populations, including pregnant women, individuals with chronic kidney disease (CKD), cancer patients, and those with nutritional deficiencies (7–9). The clinical consequences of anemia are profound, ranging from fatigue and cognitive impairment to increased morbidity and mortality, underscoring the imperative to elucidate its multifactorial pathogenesis (10, 11).

Recent advances have revealed a compelling association between alterations in gut microbial composition and the development or exacerbation of anemia. For instance, gestational anemia (GA), a frequent complication during pregnancy, has been linked to distinct gut microbiota signatures, with reduced microbial diversity and shifts in specific bacterial genera observed in affected women (2, 12). Similarly, in CKD and end-stage renal disease (ESRD) patients undergoing hemodialysis, gut dysbiosis correlates with anemia severity and responsiveness to erythropoiesis-stimulating agents, suggesting a mechanistic role of the microbiota in modulating erythropoietic pathways (5). Moreover, the gut microbiota’s influence extends to iron-deficiency anemia (IDA), where causal relationships between specific microbial taxa and IDA risk have been demonstrated using Mendelian randomization approaches, highlighting potential microbial targets for therapeutic intervention (13, 14).

The bidirectional interactions between the gut microbiota and host iron metabolism are complex. Iron availability shapes microbial community structure, while microbial metabolites and immune modulators influence iron absorption and systemic iron homeostasis (15, 16). Disruptions in this equilibrium promote dysbiosis, favoring pathogenic bacteria that exacerbate inflammation and impair nutrient absorption, thereby contributing to anemia pathogenesis (17, 18). Additionally, environmental factors such as pesticide exposure have been implicated in GA through microbiota-mediated mechanisms, further underscoring the intricate environmental-host-microbiota axis in anemia development (19). Interventions targeting the gut microbiota, including probiotics, prebiotics, dietary modifications, and fecal microbiota transplantation, have shown promise in restoring microbial balance and improving anemia outcomes in various clinical contexts, such as cancer-associated anemia and CKD-related anemia (8, 20).

Clinically, the recognition of gut microbiota alterations as contributors to anemia pathophysiology offers novel diagnostic and therapeutic avenues. Microbial signatures may serve as predictive biomarkers for anemia risk, particularly in vulnerable populations like pregnant women, enabling early identification and intervention (12). Furthermore, understanding microbiota-mediated mechanisms can inform personalized treatment strategies that complement conventional anemia therapies, potentially enhancing efficacy and minimizing adverse effects (21, 22). The integration of microbiome research into anemia management represents a paradigm shift, emphasizing the gut as a critical organ influencing hematological health.

In summary, the interplay between gut microbiota and anemia encompasses multifaceted mechanisms involving microbial composition changes, metabolic interactions, immune modulation, and environmental influences. This emerging field holds significant clinical relevance, offering insights into anemia’s etiology and unveiling microbiota-targeted interventions that may improve patient outcomes across diverse clinical settings. The present review aims to systematically delineate these mechanisms, evaluate the impact of gut microbiota on anemia, and explore their translational potential in diagnosis and therapy.

Although several reviews have addressed the relationship between gut microbiota and anemia, most have focused on single disease entities such as iron deficiency anemia or chronic kidney disease–related anemia, or on isolated mechanisms including iron metabolism or inflammation. To date, no review has comprehensively integrated gut microbiota–mediated regulation of iron metabolism, vitamin synthesis, immune modulation, and their interactions across multiple anemia phenotypes within a unified mechanistic framework.

Importantly, previous studies have largely remained descriptive and association-based, with limited critical evaluation of evidence strength, mechanistic consistency, and translational relevance.

Therefore, this review not only synthesizes current advances but also provides a structured mechanistic framework linking microbiota–metabolite–host interactions across different anemia subtypes. In addition, we critically assess the level of evidence supporting each mechanism and highlight key gaps in causal inference and clinical translation. This approach provides a more integrated and evidence-stratified perspective to guide future microbiome-based precision strategies for anemia management.

## 2 Literature search strategy

A systematic literature search was conducted using PubMed, Web of Science, Scopus, and Google Scholar to identify studies relevant to the role of gut microbiota in anemia, with particular emphasis on iron metabolism, inflammation, immune regulation, and renal anemia. The search strategy combined MeSH terms and free-text keywords, including “gut microbiota,” “intestinal microbiome,” “anemia,” “iron metabolism,” “short-chain fatty acids,” and “erythropoiesis,” using Boolean operators “AND” and “OR.” Studies published in English from January 2000 to June 2026 were included. Eligible articles comprised original experimental and clinical studies as well as high-quality reviews. Non-peer-reviewed publications, conference abstracts without full text, and studies unrelated to gut microbiota–anemia mechanisms were excluded. Additional relevant studies were identified through manual screening of reference lists from included articles.

## 3 Main body

### 3.1 Gut microbiota influence on iron absorption and utilization

The gut microbiota plays a pivotal role in modulating intestinal iron absorption and utilization, primarily through its interactions with the intestinal epithelium and luminal environment. One key mechanism involves the regulation of iron transport proteins expressed on the intestinal epithelial cells, such as divalent metal transporter 1 (DMT1) and ferroportin 1 (FPN1). These proteins are essential for the uptake of dietary iron and its export into the circulation, respectively. Gut microbes can influence the expression and activity of these transporters, thereby affecting iron absorption efficiency. For instance, certain probiotic strains, notably lactic acid bacteria like Lactobacillus species, produce organic acids that acidify the intestinal lumen. This acidification lowers the pH, which enhances the solubility of iron, particularly non-heme iron, facilitating its uptake by enterocytes. Additionally, the gut microbiota maintains a delicate balance between beneficial and pathogenic bacteria. Dysbiosis, characterized by an overgrowth of pathogenic bacteria, can lead to competition for limited iron resources in the gut lumen. Pathogens often possess high-affinity iron acquisition systems that sequester iron, thereby limiting its availability for the host and commensal microbes. This competition can impair iron absorption and increase the risk of iron deficiency anemia. Experimental studies have demonstrated that high dietary iron intake can alter the gut microbial composition, reducing beneficial bacteria such as Akkermansia, Bifidobacterium, and Lactobacillus, while increasing pathogenic taxa like Romboutsia and Erysipelatoclostridium, which may contribute to intestinal inflammation and villous damage, further impairing iron absorption. Moreover, probiotic supplementation has been shown to modulate gut microbiota composition favorably, enhancing iron bioavailability and absorption. For example, administration of multispecies probiotics in animal models increased iron content in the duodenum and liver, indicating improved iron uptake and storage. Conversely, gut microbiota dysbiosis in conditions such as chronic kidney disease and gestational anemia correlates with reduced microbial diversity and decreased abundance of beneficial bacteria, which is associated with impaired iron metabolism and anemia progression. Collectively, these findings underscore the multifaceted role of gut microbiota in regulating iron absorption and utilization via modulation of epithelial transporter expression, luminal pH, and microbial competition for iron, highlighting the potential of microbiota-targeted interventions to improve iron status and reduce anemia risk (6, 14, 23–27).

Although substantial evidence suggests that gut microbiota influences intestinal iron absorption through modulation of luminal pH, microbial competition, and epithelial iron transporters, much of the current evidence originates from animal models or *in vitro* studies. Direct evidence demonstrating microbiota-mediated regulation of DMT1 and ferroportin expression in humans remains limited. Moreover, differences in dietary iron intake, microbial composition, and host genetics may contribute to inconsistent findings across studies. Future human intervention studies are required to determine the relative contribution of specific microbial taxa to iron absorption.

### 3.2 Gut microbiota and iron metabolism-related signaling pathways

Gut microbiota exerts significant influence on systemic iron homeostasis through modulation of key signaling pathways, notably involving the hepatic hormone hepcidin, the master regulator of iron metabolism. Hepcidin controls iron egress by binding to ferroportin on enterocytes, macrophages, and hepatocytes, leading to its internalization and degradation, thereby reducing iron absorption and release from stores. The gut microbiota can indirectly regulate hepcidin expression by modulating systemic and intestinal inflammation. Microbial metabolites such as short-chain fatty acids (SCFAs), including butyrate, propionate, and acetate, have anti-inflammatory properties that can suppress pro-inflammatory cytokines like interleukin-6 (IL-6), a potent inducer of hepcidin. By attenuating inflammation, SCFAs may reduce hepcidin levels, enhancing iron absorption and mobilization. Conversely, dysbiosis characterized by an increase in pathogenic bacteria can elevate lipopolysaccharide (LPS) levels, triggering inflammatory cascades that upregulate hepcidin expression and contribute to iron sequestration and anemia of inflammation. Extracellular vesicles (EVs) from both host and microbiota are emerging as novel mediators in iron metabolism regulation. EVs can carry iron-binding proteins such as ferritin and express transferrin receptors, influencing iron transport and storage. For example, bone marrow-derived EVs induce hepcidin expression in β-thalassemia models, suggesting a role in pathological iron regulation. Although the direct involvement of microbiota-derived EVs in hepcidin modulation remains to be fully elucidated, their potential to affect macrophage iron recycling and intestinal iron absorption is an area of active research. Beyond host-derived extracellular vesicles, increasing evidence suggests that microbiota-derived extracellular vesicles (MEVs) represent an important mechanism of gut–host communication. Owing to their nanoscale size (20–400 nm), MEVs can cross the intestinal epithelial barrier through transcellular transport or via disrupted tight junctions during inflammation. Following systemic dissemination, MEVs deliver bacterial proteins, nucleic acids, lipids, and metabolites to macrophages, hepatocytes, and endothelial cells, thereby modulating inflammatory signaling and iron metabolism. Recent studies indicate that MEVs influence macrophage polarization, cytokine secretion, and hepatic immune responses, suggesting a potential role in regulating hepcidin expression and systemic iron homeostasis. However, direct evidence linking MEVs to anemia remains limited and warrants further investigation (28). Alterations in gut microbiota induced by dietary iron or disease states can reshape hepatic iron accumulation, inflammatory responses, and lipid metabolism via the microbiota-gut-liver axis, further influencing hepcidin regulation. These complex interplays highlight the gut microbiota as a critical modulator of iron metabolism-related signaling pathways, especially through hepcidin regulation and inflammatory mediators, offering potential therapeutic targets for disorders of iron homeostasis (29–33).

Although the IL-6–hepcidin axis is widely recognized as a key mechanism linking gut microbiota to systemic iron homeostasis, it is unlikely to represent the only regulatory pathway. Other microbial metabolites, extracellular vesicles, bile acids, and host metabolic signals may also participate in iron regulation, but their relative importance remains poorly understood. Furthermore, current evidence mainly demonstrates associations rather than direct mechanistic causality.

### 3.3 Role of gut microbiota in iron deficiency anemia

Iron deficiency anemia (IDA) is closely associated with alterations in gut microbiota composition and function, which contribute to impaired iron absorption and chronic inflammation and exacerbate anemia development. Clinical and experimental studies have demonstrated that patients with IDA exhibit decreased gut microbial diversity and reduced abundance of beneficial bacteria such as Bifidobacterium, Lactobacillus, and Faecalibacterium prausnitzii. These changes impair the gut barrier function and promote intestinal inflammation, which can elevate hepcidin levels, further limiting iron absorption. Dysbiosis in IDA is characterized by increased colonization of pathogenic bacteria, including members of Enterobacteriaceae, which compete with the host for iron and produce pro-inflammatory metabolites. Mendelian randomization and *in vivo* studies have identified specific microbial taxa, such as Desulfovibrio, Actinomyces, and the Ruminococcus gnavus group, that are causally linked to IDA risk, suggesting a bidirectional relationship between gut microbiota and iron status. Moreover, iron supplementation, while necessary for anemia treatment, may adversely affect the gut microbiota by increasing luminal iron availability, which favors pathogenic bacteria growth and reduces beneficial SCFA-producing microbes, potentially leading to gastrointestinal side effects and impaired microbiota recovery after antibiotic exposure. Probiotic and prebiotic interventions have shown promise in restoring microbial balance, enhancing iron absorption, and reducing inflammation in IDA. For example, supplementation with prebiotic oligosaccharides (GOS/FOS) and probiotics can increase iron bioavailability by modulating gut microbiota composition and promoting expression of iron-binding and transport proteins. Additionally, microbial metabolites such as 1,3-diaminopropane and reuterin have been identified as regulators of intestinal iron absorption through inhibition of hypoxia-inducible factor 2α (HIF-2α), a key transcription factor in iron uptake. In special populations, such as pregnant women with gestational anemia and patients with chronic kidney disease, gut microbiota dysbiosis correlates with anemia severity and response to erythropoiesis-stimulating agents, emphasizing the clinical significance of microbiota in iron deficiency states. Collectively, these findings underscore that gut microbiota dysbiosis contributes to iron absorption impairment and chronic inflammation in IDA, and that microbiota-targeted therapies may offer novel approaches to anemia management (13, 14, 20, 25, 34–36).Conventional oral iron supplementation remains the standard treatment for iron deficiency anemia but frequently increases luminal iron availability, favoring the expansion of opportunistic pathogens such as Enterobacteriaceae while reducing beneficial SCFA-producing bacteria. These alterations may aggravate intestinal inflammation and gastrointestinal adverse effects. Emerging precision nutritional strategies seek to overcome these limitations by improving iron bioavailability while preserving gut microbial homeostasis. Examples include microencapsulated iron formulations, sucrosomial^®^ iron, nanoparticle-based iron delivery systems, heme iron preparations, synbiotics, prebiotic fibers, and microbiota-directed complementary foods (MDCFs). These approaches aim to selectively enhance iron absorption, reduce luminal free iron, and minimize dysbiosis. Although preliminary studies have demonstrated improved tolerability and favorable microbiome profiles, robust randomized controlled trials remain limited (16).

Current evidence consistently demonstrates gut microbiota alterations in patients with IDA; however, whether dysbiosis is a cause or a consequence of iron deficiency remains controversial. Iron supplementation itself profoundly reshapes the gut microbiota, making it difficult to distinguish disease-associated microbial signatures from treatment-induced changes. Future longitudinal studies with treatment-naïve patients are essential to establish causality.

### 3.4 Gut microbiota’s ability to synthesize vitamin B12 and folate

Certain gut microbiota possess the enzymatic machinery to synthesize essential vitamins, notably vitamin B12 (cobalamin) and folate (vitamin B9), which serve as critical cofactors in erythropoiesis. These vitamins are indispensable for DNA synthesis and cell division, processes vital to the generation of red blood cells. The intestinal microbiota contributes to the host’s vitamin pool by producing these micronutrients, especially in the colon where bacterial density is highest. For example, specific bacterial taxa such as Bifidobacterium and Lactobacillus species have been identified as folate producers, while some Propionibacterium and certain Clostridium species contribute to cobalamin biosynthesis. The bioavailability of these microbially synthesized vitamins depends on their release and absorption in the gut, which is influenced by the composition and metabolic activity of the microbiota. Vitamin B12 biosynthesis is considerably more complex than folate synthesis because it involves more than 30 enzymatic reactions encoded by large gene clusters. Consequently, complete cobalamin biosynthesis is rarely achieved by a single bacterial species within the gut ecosystem. Instead, vitamin B12 production often depends on cooperative metabolic interactions among microbial consortia, in which different bacterial taxa exchange biosynthetic intermediates through metabolic cross-feeding. Such syntrophic relationships enhance the efficiency of cobalamin production and contribute to microbial community stability. Disruptions in the gut microbial ecosystem—termed dysbiosis—can reduce the abundance of these vitamin-producing bacteria, thereby diminishing endogenous vitamin synthesis. This reduction can be particularly consequential because vitamin B12 and folate deficiencies are well-established causes of megaloblastic anemia, characterized by impaired DNA synthesis leading to the production of abnormally large and dysfunctional red blood cells. Thus, the gut microbiota’s capacity to synthesize vitamin B12 and folate represents a key mechanistic link between intestinal microbial health and the maintenance of hematological homeostasis, underscoring the importance of microbiota integrity in preventing anemia related to vitamin deficiencies (37, 38).

Although numerous gut bacteria possess genes involved in vitamin B12 and folate biosynthesis, the actual contribution of microbiota-derived vitamins to host nutritional status remains uncertain. Since vitamin B12 is primarily absorbed in the terminal ileum whereas most microbial synthesis occurs in the colon, the physiological significance of bacterial vitamin production requires further clarification.

### 3.5 Relationship between gut microbiota dysbiosis and megaloblastic anemia

Gut microbiota dysbiosis can adversely affect the absorption and metabolism of vitamin B12 and folate, precipitating their systemic deficiency and consequent megaloblastic anemia. Dysbiosis may impair the intestinal mucosal barrier and alter the local microenvironment, thereby hindering the uptake of these vitamins from dietary and microbial sources. For instance, inflammation or damage to the intestinal epithelium, often associated with dysbiosis, can compromise intrinsic factor production or receptor function necessary for vitamin B12 absorption. Moreover, dysbiotic shifts may favor bacterial populations that compete for or degrade these vitamins, further exacerbating deficiency. Clinical studies have demonstrated that patients with altered gut microbiota profiles often exhibit reduced serum levels of vitamin B12 and folate, correlating with hematological abnormalities characteristic of megaloblastic anemia. Interventional studies have shown that modulation of the gut microbiota through probiotics or dietary changes can improve vitamin status and ameliorate anemia symptoms, highlighting the therapeutic potential of targeting microbiota to restore vitamin homeostasis. These findings emphasize that gut microbiota dysregulation is not merely a consequence but a contributory factor in the pathogenesis of megaloblastic anemia, mediated through disrupted vitamin metabolism and absorption (38–40).

Most available studies have reported associations between gut dysbiosis and vitamin deficiency; however, evidence directly linking microbiota alterations to megaloblastic anemia remains scarce. In addition, confounding factors such as gastrointestinal disorders, dietary habits, and medication use complicate interpretation of current clinical findings.

### 3.6 Potential therapeutic value of probiotic intervention for vitamin-related anemia

Probiotic supplementation emerges as a promising strategy to restore gut microbial balance, enhance vitamin synthesis, and improve absorption, thereby offering potential therapeutic benefits for vitamin-related anemia. Probiotics, including strains of Lactobacillus and Bifidobacterium, have been shown to increase the abundance of beneficial bacteria capable of producing folate and vitamin B12, as well as to modulate the intestinal environment to favor nutrient uptake. Animal models have demonstrated that probiotic administration can elevate systemic levels of these vitamins and improve hematological parameters indicative of anemia correction. Clinical trials further support these findings, reporting improved vitamin status and reduced anemia symptoms following probiotic supplementation in affected individuals. Additionally, probiotics may exert anti-inflammatory effects that help restore mucosal integrity, facilitating better absorption of vitamins. The integration of probiotics into anemia management protocols could thus complement traditional supplementation by addressing underlying microbial imbalances that impair vitamin metabolism. However, while preliminary results are encouraging, further well-designed clinical studies are warranted to establish standardized probiotic regimens, elucidate mechanisms, and confirm long-term efficacy in treating vitamin deficiency anemia (38, 39, 41).

Although probiotics have shown promising effects in improving vitamin status in experimental studies, clinical evidence remains inconsistent due to differences in bacterial strains, dosages, treatment duration, and patient populations. Large randomized controlled trials are still lacking, preventing definitive recommendations for routine clinical application.

### 3.7 Mechanisms by which gut microbiota regulates host immune system

The gut microbiota plays a pivotal role in modulating the host immune system, primarily through regulation of the intestinal mucosal immune response to maintain immune homeostasis and prevent chronic inflammation. The intestinal mucosa is a critical interface where the host immune system continuously interacts with a complex and diverse microbial community. This interaction is essential for the development and function of both innate and adaptive immunity. Gut microbiota influences immune responses by shaping the intestinal epithelial barrier, promoting the production of antimicrobial peptides, and modulating the activity of immune cells such as macrophages, dendritic cells, and T lymphocytes. Metabolites produced by gut bacteria, including short-chain fatty acids (SCFAs) like butyrate, propionate, and acetate, act as signaling molecules that regulate immune cell differentiation and cytokine production, thus influencing inflammation levels. For instance, SCFAs can enhance regulatory T cell (Treg) differentiation and suppress pro-inflammatory cytokines, contributing to immune tolerance (1).Mechanistically, SCFAs regulate immune homeostasis through both epigenetic and receptor-dependent pathways. Butyrate and propionate act as endogenous histone deacetylase (HDAC) inhibitors, increasing histone acetylation at the Foxp3 promoter and conserved non-coding sequence regions, thereby promoting stable differentiation and expansion of regulatory T (Treg) cells. In parallel, SCFAs activate G-protein-coupled receptors, particularly GPR43 (FFAR2), GPR41 (FFAR3), and GPR109A, which are expressed on intestinal epithelial cells, dendritic cells, and macrophages. Activation of these receptors stimulates IL-10 and TGF-β production while suppressing NF-κB activation, thereby reducing the secretion of pro-inflammatory cytokines including IL-6, IL-1β, and TNF-α. These coordinated effects contribute to immune tolerance, attenuate chronic inflammation, and indirectly improve erythropoiesis through modulation of the IL-6–hepcidin axis (42). Gut microbiota-derived microbial-associated molecular patterns (MAMPs) activate innate immune signaling through pattern-recognition receptors (PRRs). Lipopolysaccharide (LPS) derived from Gram-negative bacteria binds Toll-like receptor 4 (TLR4), triggering recruitment of the adaptor protein MyD88 and sequential activation of IRAK1/4, TRAF6, and TAK1. This signaling cascade activates NF-κB and MAPK pathways, resulting in increased transcription of IL-6, TNF-α, and IL-1β. Elevated circulating IL-6 subsequently activates hepatic JAK2/STAT3 signaling, inducing hepcidin expression. Increased hepcidin promotes ferroportin internalization and degradation on enterocytes and macrophages, leading to iron sequestration, impaired intestinal iron absorption, and functional iron deficiency characteristic of anemia of inflammation. Dysbiosis, or imbalance in gut microbial composition, can disrupt this equilibrium, leading to aberrant immune responses and chronic inflammation. In the context of anemia, the gut microbiota’s regulation of immune homeostasis is crucial because chronic inflammation mediated by immune dysregulation often underlies anemia of inflammation. Studies have demonstrated that specific bacterial taxa and their metabolites modulate macrophage and T cell functions, which are central to inflammatory processes influencing iron metabolism and erythropoiesis. Therefore, the gut microbiota maintains immune homeostasis by modulating mucosal immunity, regulating immune cell function via microbial metabolites, and preventing chronic inflammation that can contribute to anemia development (42–46).

Current evidence supports an important role for gut microbiota in immune homeostasis; however, immune responses are highly context-dependent and influenced by multiple host factors. The specific microbial taxa responsible for regulating immune-mediated erythropoiesis remain poorly defined, and mechanistic studies are still limited.

### 3.8 Relationship between chronic inflammation and anemia

Chronic inflammation is a well-recognized contributor to anemia, particularly anemia of inflammation (AI), which is prevalent in chronic diseases such as chronic kidney disease, cancer, and infections. Inflammatory cytokines, notably interleukin-6 (IL-6), play a central role by inducing the hepatic production of hepcidin, a key regulator of systemic iron homeostasis (47). Elevated hepcidin levels inhibit iron export from macrophages and enterocytes by degrading the iron transporter ferroportin, leading to iron sequestration and hypoferremia despite adequate or increased iron stores. This iron-restricted erythropoiesis results in decreased hemoglobin synthesis and anemia. Furthermore, chronic inflammation suppresses erythropoietin production and impairs erythroid progenitor responsiveness, exacerbating anemia. The gut microbiota is implicated in modulating systemic and intestinal inflammation; dysbiosis can exacerbate gut barrier dysfunction and promote translocation of microbial products, fueling systemic inflammatory responses. For example, in chronic kidney disease and cancer-associated anemia, altered gut microbiota composition correlates with increased inflammation and worsened anemia. Additionally, gut microbial metabolites can influence immune cell activation and cytokine profiles, further modulating inflammation. Studies have reported that gut microbiota dysbiosis in anemia patients is associated with increased inflammatory markers such as C-reactive protein (CRP) and pro-inflammatory cytokines, which contribute to the pathogenesis of anemia. Therefore, chronic inflammation mediated by immune activation and gut microbiota dysbiosis leads to functional iron deficiency and impaired erythropoiesis, underscoring the intertwined relationship between inflammation and anemia (6, 8, 20, 48, 49).

Although activation of the IL-6–hepcidin pathway is considered a hallmark of anemia of inflammation, inflammation-related anemia is a multifactorial process involving impaired erythropoietin production, shortened erythrocyte lifespan, and bone marrow suppression. Therefore, focusing solely on microbiota-induced inflammation may oversimplify disease pathogenesis.

### 3.9 Potential of gut microbiota modulation to regulate immune responses and improve anemia

Modulating the gut microbiota presents a promising strategy to regulate immune responses and ameliorate anemia, particularly anemia linked to chronic inflammation. Interventions such as probiotics, prebiotics, fecal microbiota transplantation (FMT), and dietary modifications have been shown to restore microbial balance, reduce systemic inflammation, and improve nutrient absorption, all of which are critical for effective erythropoiesis (43, 50). Probiotics can enhance gut barrier integrity, suppress pathogenic bacteria, and promote the production of anti-inflammatory metabolites like SCFAs, which in turn modulate immune cell function and cytokine production. Clinical studies have demonstrated that probiotic supplementation can increase regulatory T cell populations and reduce pro-inflammatory cytokines, thereby attenuating chronic inflammation associated with anemia. FMT has emerged as a powerful tool to reconstitute a healthy microbiome, showing potential for improving immune-related anemia by restoring microbial diversity and function. In patients with cancer-associated anemia, microbiome-focused therapies have been linked to improved responses to chemotherapy and reduced inflammation. Moreover, in chronic kidney disease and gestational anemia, gut microbiota-targeted interventions have shown benefits in reducing inflammation and improving hematological parameters. Novel approaches, including engineered probiotics and bacteriophage therapies, offer precision modulation of the gut microbiome to optimize immune regulation. Collectively, these microbiome-centered interventions hold significant potential to improve anemia outcomes by dampening inflammatory pathways, enhancing iron metabolism, and supporting hematopoiesis. Future research integrating microbiota modulation with conventional anemia treatments could lead to innovative, effective clinical strategies (8, 45, 51–54).

Despite encouraging preliminary findings, microbiota-targeted therapies remain at an early stage of clinical translation. Most published studies involve small sample sizes, heterogeneous interventions, and short follow-up periods. Moreover, standardized protocols for probiotics, prebiotics, and fecal microbiota transplantation are lacking, limiting reproducibility and preventing evidence-based clinical recommendations.

Based on the above mechanisms, distinct gut microbiota alterations and pathways are associated with different anemia phenotypes, as summarized in **Table 1**.

**Table 1:** mechanistic links between gut microbiota and different types of anemia.

Anemia type	Key gut microbiota changes	Key mechanisms	Clinical/experimental evidence	Intervention strategies	References
Iron Deficiency Anemia (IDA)	↓Bifidobacterium, Lactobacillus, Faecalibacterium prausnitzii; ↑Enterobacteriaceae, Desulfovibrio, Ruminococcus gnavus	Competition for luminal iron; increased intestinal inflammation; elevated hepcidin; impaired iron absorption	Mendelian randomization studies; clinical cohorts; animal models	Probiotics/prebiotics; iron supplementation; dietary modulation	(11–13, 23, 31–33)
Anemia of Inflammation (AI)	Reduced microbial diversity; ↑LPS-producing bacteria (e.g., Enterobacteriaceae)	IL-6–hepcidin axis activation; ferroportin degradation; iron sequestration; suppressed erythropoietin response	CKD patient studies; cancer-associated anemia studies	Probiotics; fecal microbiota transplantation (FMT); anti-inflammatory treatment; dietary modulation	(1, 6, 11, 43, 44)
Vitamin B12/Folate Deficiency Anemia	↓Vitamin-producing bacteria (Bifidobacterium, Propionibacterium, certain Clostridium species)	Impaired microbial vitamin synthesis; reduced intestinal absorption; DNA synthesis disruption; megaloblastic anemia	Clinical observations; probiotic intervention studies	Probiotics/prebiotics; vitamin supplementation	(35–37)
Renal Anemia (CKD-related)	Gut dysbiosis; ↓SCFA-producing bacteria (e.g., Faecalibacterium, Roseburia)	Chronic inflammation; impaired erythropoietin response; iron misdistribution; reduced erythropoiesis	Hemodialysis patient cohorts; CKD clinical cohorts	Probiotics/prebiotics; SCFA supplementation; erythropoietin therapy	(3, 11, 48)

*↑ indicates increase;↓ indicates decrease compared with healthy controls.

Although distinct microbial signatures have been reported across different anemia phenotypes, substantial overlap exists among disease-specific microbial alterations. Therefore, caution should be exercised when interpreting individual bacterial taxa as disease-specific biomarkers, and functional characterization may be more informative than taxonomic composition alone.

Microbiota-targeted interventions have emerged as promising adjunctive strategies for anemia management, with representative approaches summarized in **Table 2**.

**Table 2:** Microbiota-targeted interventions for anemia: evidence and limitations.

**Intervention**	**Target mechanism**	**Evidence strength**	**Limitations**	**References**
Probiotics (Lactobacillus, Bifidobacterium)	↑ iron solubility; ↑ SCFAs; ↓ inflammation	Animal models and small clinical trials	High strain specificity; small sample sizes; variable efficacy	(37, 40, 44)
Prebiotics (GOS/FOS)	Promote beneficial bacteria; ↑ iron absorption	Experimental and preliminary clinical data	GI side effects in some patients	(37, 40)
Dietary modulation	Alters microbiota–iron interaction	Observational and small intervention studies	Adherence variability; lack of RCTs	(16, 23)
Fecal microbiota transplantation (FMT)	Restores microbial diversity	Case reports /small-scale trials	Standardized safety protocols; unknown long-term effects	(20, 49)
Combined iron + microbiota therapy	Reduces iron-induced dysbiosis	Preclinical studies	Lack of large RCTs; optimal combination unknown	(25, 29)

*↑ indicates increase; ↓ indicates decrease compared with healthy controls.

Evidence strength is graded based on study design (RCT > cohort/case-control > case report > animal study).

Collectively, these interventions demonstrate considerable therapeutic potential; however, current evidence remains insufficient to support routine clinical implementation. Future studies should prioritize standardized intervention protocols, strain-specific evaluation, and long-term safety assessment to facilitate translation into precision medicine.

## 4 Conclusion

The relationship between gut microbiota alterations and anemia is increasingly recognized as bidirectional and context-dependent. Current evidence suggests three possible scenarios: (1) gut dysbiosis as a causal factor contributing to anemia through impaired nutrient absorption, immune activation, and metabolic dysregulation; (2) anemia as a driver of microbiota alterations via changes in iron availability, inflammation, and host physiology; and (3) a bidirectional feedback loop in which microbiota and host hematological status mutually influence each other.

Among these, causal evidence is strongest for iron metabolism–related pathways supported by Mendelian randomization and animal models, whereas evidence for vitamin-mediated and immune-mediated mechanisms remains largely associative.

Disentangling causality from correlation is therefore essential for translating microbiome findings into clinical interventions, particularly in the context of microbiota-targeted therapies.

In conclusion, the intricate interplay between the gut microbiota and anemia represents a rapidly evolving frontier in medical research, with profound implications for understanding and managing this widespread condition. From an expert perspective, it is clear that the gut microbiota exerts a pivotal influence on key physiological processes such as iron metabolism, vitamin synthesis, and immune modulation—each of which directly impacts the onset and progression of anemia. This multifaceted role underscores the importance of viewing anemia not merely as a hematologic disorder but as a systemic condition intricately linked to microbial ecology within the gastrointestinal tract.

Conventional oral iron supplementation, particularly ferrous salts, is effective but may induce gut microbiota dysbiosis by increasing luminal free iron, which promotes the expansion of opportunistic pathogens such as Enterobacteriaceae and reduces beneficial SCFA-producing bacteria. In contrast, emerging precision nutritional strategies aim to improve iron bioavailability while minimizing microbiota disruption. These include microencapsulated and sucrosomial iron formulations that control iron release and enhance absorption, heme iron preparations with higher bioavailability, and microbiota-directed complementary foods (MDCFs) or synbiotic approaches that combine iron supplementation with microbiota modulation. Collectively, compared with conventional iron salts, these strategies reduce iron-induced dysbiosis while improving iron utilization, representing a more microbiota-friendly approach for managing iron deficiency anemia.

The current body of evidence highlights that dysbiosis, or imbalance in the gut microbial community, contributes to anemia through diverse mechanisms. Particularly in iron deficiency anemia and vitamin deficiency anemia, alterations in microbial composition can impair nutrient absorption, disrupt metabolic pathways, and exacerbate inflammatory responses, thereby aggravating hematologic deficits. However, while numerous studies have delineated associations between specific microbial patterns and anemia phenotypes, the challenge remains to establish definitive causal relationships. This necessitates rigorous longitudinal and mechanistic investigations that can disentangle correlation from causation and identify precise microbial targets for intervention.

Balancing the various research perspectives requires an integrative approach that considers host genetics, dietary factors, environmental influences, and microbial interactions. Such a holistic framework will facilitate the identification of biomarkers predictive of anemia risk and therapeutic responsiveness. Moreover, it will enable the development of personalized treatment modalities that harness the gut microbiota’s capacity to modulate iron homeostasis and immune function.

Looking forward, the translation of these insights into clinical practice holds great promise. Microecological interventions, including probiotic supplementation and fecal microbiota transplantation, emerge as innovative strategies to restore microbial equilibrium and ameliorate anemia symptoms (50). These approaches have the potential to complement conventional therapies by enhancing nutrient bioavailability and reducing inflammation, ultimately improving patient outcomes and quality of life. Nonetheless, although microbiota-targeted interventions such as probiotics, prebiotics, dietary modulation, and fecal microbiota transplantation show promise in experimental and early clinical studies, their translational application in anemia management remains limited. Major challenges include high inter-individual variability in microbiome composition, strain-specific and non-reproducible effects of probiotics, lack of standardized treatment protocols, and insufficient long-term safety data. Furthermore, most clinical studies are constrained by small sample sizes and heterogeneous study designs, limiting generalizability. At present, microbiota-based therapies should be considered adjunctive rather than replacement strategies for conventional anemia treatment. Future progress will likely depend on precision microbiome profiling, patient stratification, and integration of multi-omics approaches to enable personalized interventions.

In summary, advancing our understanding of the gut microbiota’s role in anemia offers a transformative avenue for both research and therapy. By integrating multidisciplinary expertise and leveraging cutting-edge technologies, future studies can unlock novel diagnostic and therapeutic paradigms that transcend traditional boundaries. This balanced and nuanced perspective not only enriches the scientific discourse but also paves the way for more effective, microbiota-informed management of anemia in diverse patient populations.

## 5 Limitations

This review has several limitations. First, it is a narrative rather than a fully systematic review, which may introduce selection bias. Second, substantial heterogeneity exists among included studies in terms of experimental design, disease models, and patient populations, which may limit direct comparability. Third, although accumulating evidence suggests a close association between gut microbiota dysbiosis and anemia, much of the mechanistic insight is derived from preclinical studies, and robust longitudinal clinical evidence remains limited. In addition, publication bias toward positive findings cannot be excluded. Therefore, the conclusions should be interpreted with caution, particularly regarding causality between gut microbiota alterations and anemia development.

## 6 Future directions

Future studies should aim to clarify the causal relationship between gut microbiota and anemia through large-scale, multicenter longitudinal clinical cohorts. Integrative multi-omics approaches, including metagenomics, metabolomics, and transcriptomics, are needed to elucidate functional interactions between microbial communities and host pathways involved in iron metabolism, immune regulation, and erythropoiesis. Further mechanistic investigations should focus on microbiota-derived metabolites, particularly short-chain fatty acids, in the regulation of renal anemia and systemic inflammation. From a translational perspective, microbiota-targeted interventions such as probiotics, prebiotics, dietary modulation, and fecal microbiota transplantation warrant further clinical validation. Ultimately, personalized microbiome-based therapies may offer promising strategies for precision treatment of anemia.

## References

[1] Zhang S Wu Z Zhang S Ru Y Wang Q Tong H . The intricate microbial-gut-brain axis in Alzheimer's disease: a review of microbiota-targeted strategies. Food Funct. (2025) 16:8320–44. doi: 10.1039/d5fo03139g 41085348

[2] Long Y Liang F Guo R Zhu C Zhao X Wang X . Gut microbiota signatures in gestational anemia. Front Cell Infect Microbiol. (2021) 11:549678. doi: 10.3389/fcimb.2021.549678 33718259 PMC7947918

[3] Mandell J Caulfield LE Talegawkar S Mathad JS Naik S Alexander M . Maternal anemia during pregnancy and its association with the gut microbiota profile in pregnant and postpartum women, and their infants: a cohort study. Am J Clin Nutr. (2025) 122:1669–78. doi: 10.1016/j.ajcnut.2025.09.035 41015193 PMC12574691

[4] He Y Hu H Liang X Liang J Li F Zhou X . Gut microbes-muscle axis in muscle function and meat quality. Sci China Life Sci. (2025). doi: 10.1007/s11427-024-2885-4 40220074

[5] Zhu Y Tang Y He H Hu P Sun W Jin M . Gut microbiota correlates with clinical responsiveness to erythropoietin in hemodialysis patients with anemia. Front Cell Infect Microbiol. (2022) 12:919352. doi: 10.3389/fcimb.2022.919352 35937691 PMC9355670

[6] Wang H Xue W Cheng J He Y Song Y Hu D . Altered fecal microbial and metabolic profiles reveal potential mechanisms underlying anemia in patients with chronic renal failure. Microbiol Spectr. (2025) 13:e0316624. doi: 10.1681/asn.2025g3cqvpv3 40525868 PMC12323308

[7] Zhu Y Tang Y He H Hu P Sun W Jin M . Intestinal microbiota dataset revealed by high-throughput sequencing of 16S rRNA in children with anemia in southern Peru. Data Brief. (2024) 55:110681. doi: 10.1016/j.dib.2024.110681 39081489 PMC11287011

[8] Bangolo A Amoozgar B Habibi M Simms E Nagesh VK Wadhwani S . Exploring the gut microbiome's influence on cancer-associated anemia: mechanisms, clinical challenges, and innovative therapies. World J Gastrointest Pharmacol Ther. (2025) 16:105375. doi: 10.4292/wjgpt.v16.i2.105375 40575364 PMC12188820

[9] Hain D Bednarski D Cahill M Dix A Foote B Haras MS . Iron-deficiency anemia in CKD: a narrative review for the kidney care team. Kidney Med. (2023) 5:100677. doi: 10.1016/j.xkme.2023.100677 37415621 PMC10319843

[10] Arynov A Kaidarova D Kabon B . Alternative blood transfusion triggers: a narrative review. BMC Anesthesiol. (2024) 24:71. doi: 10.1186/s12871-024-02447-3 38395758 PMC10885388

[11] Narang D Singh T Sinha R Goswami P Kumar S Sahoo N . Diabetes and anaemia: a myth or reality. J Pharm Bioallied Sci. (2024) 16:S2279–s2281. doi: 10.4103/jpbs.jpbs_213_24 39346437 PMC11426576

[12] Wei H Deng S Qin Y Yang X Chen T Wang X . Insight into the potential value of gut microbial signatures for prediction of gestational anemia. Front Cell Infect Microbiol. (2021) 11:734561. doi: 10.3389/fcimb.2021.734561 34527605 PMC8437374

[13] Lei W Liu Z Lai HP Fu R . Gut microbiota and risk of iron deficiency anemia: a two-sample Mendelian randomization study. Med (Baltimore). (2025) 104:e41617. doi: 10.1097/md.0000000000041617 39993092 PMC11856941

[14] Zhou H Fan Z Da Y Liu X Wang C Zhang T . Causal relationships between iron deficiency anemia, gut microbiota, and metabolites: insights from Mendelian randomization and *in vivo* data. Biomedicines. (2025) 13:200–7. doi: 10.3390/biomedicines13030677 40149653 PMC11940133

[15] Mayneris-Perxachs J Moreno-Navarrete JM Fernández-Real JM . The role of iron in host-microbiota crosstalk and its effects on systemic glucose metabolism. Nat Rev Endocrinol. (2022) 18:683–98. doi: 10.1038/s41574-022-00721-3 35986176

[16] Sun B Tan B Zhang P Zhu L Wei H Huang T . Iron deficiency anemia: a critical review on iron absorption, supplementation and its influence on gut microbiota. Food Funct. (2024) 15:1144–57. doi: 10.1039/d3fo04644c 38235788

[17] Zhang Q Wu W Guo F Li J Jin Y Cai G . Characteristics of gut microbiota and fecal metabolites in patients with colorectal cancer-associated iron deficiency anemia. Microorganisms. (2024) 12:14972–9. doi: 10.3390/microorganisms12071319 39065088 PMC11279063

[18] Kalteren WS Mebius MJ Verhagen EA Tanis JC Kooi EMW Bos AF . Neonatal hemoglobin levels in preterm infants are associated with early neurological functioning. Neonatology. (2021) 118:593–9. doi: 10.1159/000518655 34515185

[19] Dong C Yu S Deng S Xia Z Rigoberto FC Sultan M . Pesticide exposure induces risks of gestational anemia by maternal gut microbiota: a prospective cohort study. J Hazard Mater. (2025) 494:138465. doi: 10.1016/j.jhazmat.2025.138465 40339373

[20] Coll E Cigarran S Portolés J Cases A . Gut dysbiosis and its role in the anemia of chronic kidney disease. Toxins (Basel). (2024) 16:515–21. doi: 10.3390/toxins16110495 39591250 PMC11598790

[21] Wan D Liang X Yang L He D Du Q Zhang W . Integration of gut microbiota and metabolomics for the hematopoiesis of Siwu paste on anemia rats. Heliyon. (2023) 9:e18024. doi: 10.1016/j.heliyon.2023.e18024 37449126 PMC10336798

[22] Bianchi VE von Haehling S . The treatment of chronic anemia in heart failure: a global approach. Clin Res Cardiol. (2024) 113:1117–36. doi: 10.1007/s00392-023-02275-4 37660308

[23] Xiong Q Zhao J Tian C Ma W Miao L Liang L . Regulation of a high-iron diet on lipid metabolism and gut microbiota in mice. Anim (Basel). (2022) 12:1320–5. doi: 10.3390/ani12162063 36009656 PMC9405328

[24] Aksoyalp Z Temel A Erdogan BR . Iron in infectious diseases friend or foe?: the role of gut microbiota. J Trace Elem Med Biol. (2023) 75:127093. doi: 10.1016/j.jtemb.2022.127093 36240616

[25] Cuisiniere T Calvé A Fragoso G Oliero M Hajjar R Gonzalez E . Oral iron supplementation after antibiotic exposure induces a deleterious recovery of the gut microbiota. BMC Microbiol. (2021) 21:259. doi: 10.1186/s12866-021-02320-0 34583649 PMC8480066

[26] Zhang XL Zhou YR Xu SS Xu S Xiong YJ Xu K . Characterization of gut microbiota compositions along the intestinal tract in CD163/pAPN double knockout piglets and their potential roles in iron absorption. Microbiol Spectr. (2023) 11:e0190622. doi: 10.1128/spectrum.01906-22 36625575 PMC9927099

[27] Skrypnik K Bogdański P Schmidt M Suliburska J . The effect of multispecies probiotic supplementation on iron status in rats. Biol Trace Elem Res. (2019) 192:234–43. doi: 10.1007/s12011-019-1658-1 30746586 PMC6820595

[28] Liu Y Pan R Ouyang Y Gu W Xiao T Yang H . Pyroptosis in health and disease: mechanisms, regulation and clinical perspective. Signal Transduct Target Ther. (2024) 9:245. doi: 10.1038/s41392-024-01958-2 39300122 PMC11413206

[29] Daou Y Falabrègue M Pourzand C Peyssonnaux C Edeas M . Host and microbiota derived extracellular vesicles: crucial players in iron homeostasis. Front Med (Lausanne). (2022) 9:985141. doi: 10.3389/fmed.2022.985141 36314015 PMC9606470

[30] Xiao L Tang R Wang J Wan D Yin Y Xie L . Gut microbiota bridges the iron homeostasis and host health. Sci China Life Sci. (2023) 66:1952–75. doi: 10.1007/s11427-022-2302-5 37515687

[31] Ahmadi Badi S Bereimipour A Rohani P Khatami S Siadat SD . Interplay between gut microbiota and the master iron regulator, hepcidin, in the pathogenesis of liver fibrosis. Pathog Dis. (2024) 82:27–37. doi: 10.1093/femspd/ftae005 38555503 PMC10990161

[32] Han Y Zhang Y Chen J Jiang S Zheng Y Xu Y . Iron overload exacerbates metabolic dysfunction-associated steatohepatitis via the microbiota-gut-liver axis through lipopolysaccharide-mediated Akr1b8 activation. Free Radic Biol Med. (2025) 233:196–208. doi: 10.1016/j.freeradbiomed.2025.03.039 40157463

[33] Li H . Iron and the intestinal microbiome. Adv Exp Med Biol. (2025) 1480:345–60. doi: 10.1007/978-3-031-92033-2_22 40603801

[34] Sun B Tan B Zhang P Huang T Wei H Li C . Effects of hemoglobin extracted from Tegillarca granosa on the gut microbiota in iron deficiency anemia mice. Food Funct. (2023) 14:7040–52. doi: 10.1016/j.foodres.2022.112031 37449470

[35] Song J Zhou B Kan J Liu G Zhang S Si L . Gut microbiota: linking nutrition and perinatal depression. Front Cell Infect Microbiol. (2022) 12:932309. doi: 10.3389/fcimb.2022.932309 36093196 PMC9459161

[36] Du J Zhao X Ding X Han Q Duan Y Ren Q . The role of the gut microbiota in complications among hemodialysis patients. Microorganisms. (2024) 12:22–8. doi: 10.20944/preprints202408.0153.v1 PMC11434415 39338552

[37] Li H Ye F Li Z Peng X Wu L Liu Q . The response of gut microbiota to arsenic metabolism is involved in arsenic-induced liver injury, which is influenced by the interaction between arsenic and methionine synthase. Environ Int. (2024) 190:108824. doi: 10.1016/j.envint.2024.108824 38917623

[38] Zakrzewska Z Zawartka A Schab M Martyniak A Skoczeń S Tomasik PJ . Prebiotics, probiotics, and postbiotics in the prevention and treatment of anemia. Microorganisms. (2022) 10:1330. doi: 10.3390/microorganisms10071330 35889049 PMC9317605

[39] Gambioli R Lepore E Biondo FG Bertolani L Unfer V . Risks and limits of bariatric surgery: old solutions and a new potential option. Eur Rev Med Pharmacol Sci. (2023) 27:5831–40. doi: 10.26355/eurrev_202306_32822 37401320

[40] Aumpan N Mahachai V Vilaichone RK . Management of Helicobacter pylori infection. JGH Open. (2023) 7:3–15. doi: 10.1002/jgh3.12843 36660052 PMC9840198

[41] Hu J Guo P Mao R Ren Z Wen J Yang Q . Gut microbiota signature of obese adults across different classifications. Diabetes Metab Syndr Obes. (2022) 15:3933–47. doi: 10.2147/dmso.s387523 36601354 PMC9807070

[42] Ding G Yang X Li Y Wang Y Du Y Wang M . Gut microbiota regulates gut homeostasis, mucosal immunity and influences immune-related diseases. Mol Cell Biochem. (2025) 480:1969–81. doi: 10.1007/s11010-024-05077-y 39060829

[43] Su M Tang T Tang W Long Y Wang L Liu M . Astragalus improves intestinal barrier function and immunity by acting on intestinal microbiota to treat T2DM: a research review. Front Immunol. (2023) 14:1243834. doi: 10.3389/fimmu.2023.1243834 37638043 PMC10450032

[44] Wang J He M Yang M Ai X . Gut microbiota as a key regulator of intestinal mucosal immunity. Life Sci. (2024) 345:122612. doi: 10.1016/j.lfs.2024.122612 38588949

[45] Wang X Zhang P Zhang X . Probiotics regulate gut microbiota: an effective method to improve immunity. Molecules. (2021) 26:837–40. doi: 10.3390/molecules26196076 34641619 PMC8512487

[46] Negi S Das DK Pahari S Nadeem S Agrewala JN . Potential role of gut microbiota in induction and regulation of innate immune memory. Front Immunol. (2019) 10:2441. doi: 10.3389/fimmu.2019.02441 31749793 PMC6842962

[47] Ren W Xia Y Chen S Wu G Bazer FW Zhou B . Glutamine metabolism in macrophages: a novel target for obesity/type 2 diabetes. Adv Nutr. (2019) 10:321–30. doi: 10.1093/advances/nmy084 30753258 PMC6416106

[48] Conde Díez S de Las Cuevas Allende R Conde García E . Anemia of inflammation and iron metabolism in chronic diseases. Rev Clin Esp (Barc). (2024) 224:598–608. doi: 10.1016/j.rceng.2024.09.002 39236980

[49] Marques O Weiss G Muckenthaler MU . The role of iron in chronic inflammatory diseases: from mechanisms to treatment options in anemia of inflammation. Blood. (2022) 140:2011–23. doi: 10.1182/blood.2021013472 35994752

[50] Zhang C Li H Xu M Pu C Zhu Y Du L . Remodeling the gut ecosystem and its mechanism: natural products in the treatment of ulcerative colitis via regulating gut microbiota and metabolites. Int Immunopharmacol. (2026) 174:116378. doi: 10.1016/j.intimp.2026.116378 41713018

[51] Jiang Y Cui W Zhang Y Wang T Zheng X Li H . Fg-4592 relieves diabetic kidney disease severity by influencing metabolic profiles via gut microbiota reconstruction in both human and mouse models. Front Physiol. (2023) 14:1195441. doi: 10.3389/fphys.2023.1195441 37654676 PMC10465800

[52] Yoo JS Oh SF . Unconventional immune cells in the gut mucosal barrier: regulation by symbiotic microbiota. Exp Mol Med. (2023) 55:1905–12. doi: 10.1038/s12276-023-01088-9 37696893 PMC10545787

[53] Kant SB Bashir R Khan B Shabbir NA Nizami AA Akbar A . A cross-sectional study of the maternal health factors: the interplay between breastfeeding patterns, gut microbiota, anemia, and cardiovascular risk in lactating mothers. Cureus. (2024) 16:e76316. doi: 10.7759/cureus.76316 39850157 PMC11756575

[54] Liang Y Chen Y Lin Y Huang W Qiu Q Sun C . The increased tendency for anemia in traditional Chinese medicine deficient body constitution is associated with the gut microbiome. Front Nutr. (2024) 11:1359644. doi: 10.3389/fnut.2024.1359644 39360281 PMC11445043

